# The Feedback of Stress Phytohormones in *Avena sativa* (L.) on Soil Multi-Contamination

**DOI:** 10.3390/plants14162554

**Published:** 2025-08-16

**Authors:** Veronika Zemanová, Milan Pavlík, Milan Novák, Daniela Pavlíková

**Affiliations:** 1 Czech Agrifood Research Center, Division of Crop Management Systems, 16100 Prague, Czech Republic; 2 Department of Agroenvironmental Chemistry and Plant Nutrition, Faculty of Agrobiology, Food and Natural Resources, Czech University of Life Sciences Prague, 16500 Prague, Czech Republic

**Keywords:** abscisic acid, jasmonic acid, oat, potentially toxic elements, salicylic acid, stress response

## Abstract

As chemical messengers, phytohormones can enhance the tolerance of plants to stress caused by toxic elements (TEs) such as cadmium (Cd), lead (Pb), and zinc (Zn). This study investigated the combined toxicity of Cd, Pb, and Zn, and its impact on stress phytohormones (jasmonates, salicylic acid, and abscisic acid), in oat (*Avena sativa* L.) using anthropogenically contaminated soil in a 4-week pot experiment. The uptake of TEs by the roots increased in the multi-contaminated soil, while Zn was the only TE to be translocated to the leaves. The toxic effect of the TEs was assessed in terms of plant growth, revealing a decline in leaf dry biomass, whereas the impact on the roots was insignificant. These findings align with the levels of stress phytohormones. An increase in bioactive forms of stress phytohormones in leaves due to TEs indicates TE toxicity and leaf sensitivity. Conversely, low levels of these phytohormones, along with crosstalk between them, suggest reduced defense against TEs in the roots. The abundance of stress phytohormones declined in the following order: salicylic acid > jasmonates > abscisic acid. These results help to understand the mechanism by which plants respond to TEs, particularly their combined toxicity.

## 1. Introduction

Toxic elements (TEs) are always present in the natural environment; however, their quantities are significantly increased by anthropogenic activities. These elements are non-degradable and, consequently, accumulate in soils [1]. Environmental pollution from mining, ore smelting, and processing often occurs locally and is a critical issue [2]. The Příbram region, located about 60 km southwest of Prague, the capital of the Czech Republic, is one of the most heavily polluted areas in the country. Mining and lead processing in this area have led to increased soil contamination, primarily with lead (Pb), cadmium (Cd), and zinc (Zn), due to the high concentrations of these elements in the parent rock [3]. The elevated content of TEs in soil can be phytotoxic, disrupting physiological and biochemical processes in plants. This can affect pigment synthesis, photosynthesis, gas exchange parameters, and water regulation, as well as leading to the inactivation and denaturation of enzymes and the inhibition of functional groups in metabolically essential molecules. It can also cause disturbances in hormonal balance and N and C metabolism [4,5,6].

Plants have evolved complex adaptive systems in response to stressful environmental conditions. These systems include changes in the levels and ratios of plant hormones, which play a key role in morphological plasticity in plants [7,8]. Phytohormones promote tolerance to TEs in plants. These signaling molecules are essential for regulating various physiological and biochemical processes. While auxins (indole-3-acetic acid, IAA), gibberellins (GAs), and cytokinins (CKs) are growth-promoting hormones, abscisic acid (ABA), salicylic acid (SA), and jasmonic acid (JA) play significant roles in the stress response and adaptation of plants, often inhibiting growth [9,10]. Stress hormones can significantly affect stomatal closure, thereby influencing photosynthetic efficiency. While stomatal closure is important for regulating water loss and CO_2_ diffusion, it negatively impacts the net photosynthesis rate by decreasing PSII activity, C fixation, and the activity of photosynthetic enzymes. This ultimately results in reduced leaf area and crop yield [11].

Salicylic acid is a phenolic phytohormone that plays a crucial role in enhancing plant tolerance to abiotic stress as a signaling molecule [12,13,14,15]. It increases tolerance to stress by enhancing the activities of antioxidant enzymes involved in H_2_O_2_ detoxification and positively influencing the regulation of ion channels and photosynthetic processes [16,17]. SA is an essential regulator of photosynthesis, affecting chlorophyll content, stomatal conductance, and the activity of enzymes related to photosynthesis in plants. It enhances photosynthetic efficiency and improves the photosynthetic apparatus under TEs. SA promotes the formation of antioxidant compounds by interacting with other plant hormones, such as GA, ABA, and auxins [18]. The interaction between SA and JAs is crucial in regulating plant growth under stressful conditions. The signaling pathways of SA and JA operate antagonistically, mediated by the mitogen-activated protein kinase signaling pathway [13].

Jasmonic acid and its conjugates, such as methyl jasmonate (JA-Me) and jasmonoyl-isoleucine (JA-Ile), are collectively known as jasmonates (JAs). These fatty acid-derived compounds play regulatory roles throughout the entire plant life cycle [19]. Jasmonates are crucial in mediating responses to TE stress, acting as signals that regulate the expression of various tolerance-associated genes [20]. They limit TE accumulation while enhancing detoxification processes by coordinating the membrane transport, antioxidant enzyme activity, and chelating capacity in plants. The results of Che et al. [21] suggest that Ca^2+^ signaling may be involved in the stress-induced production of JAs.

Abscisic acid is a phytohormone that plays a crucial role in plant responses to abiotic stress and in initiating resistance to the toxicity of TEs such as Pb, Cd, and arsenic (As). Hu et al. [22] confirmed that the content of ABA increases following exposure of plants to TEs. According to these authors, this phytohormone is therefore involved in defensive mechanisms against TE toxicity. Under TE stress, ABA significantly enhances the activities of antioxidant enzymes, including superoxide dismutase, catalase, and ascorbate peroxidase. These enzymes scavenge excess reactive oxygen species (ROS) and protect cell membranes, nucleic acids, and proteins from oxidative damage [23]. An increase in ABA levels can also lead to elevated levels of non-enzymatic antioxidants, such as ascorbic acid, glutathione tripeptide, carotenoids, and α-tocopherol [24]. Furthermore, ABA plays a significant role in TE chelation by enhancing phytochelatin synthase gene expression [25]. Zhao et al. [23] stated that ABA regulates transpiration by modulating stomatal aperture. This activates complex signaling pathways in guard cells, which are mediated by kinases/phosphatases, secondary messengers, and ion channel regulation. The size of the stomatal aperture is affected by both Ca^2+^-dependent and -independent pathways [26]. Reduced transpiration results in decreased uptake and transport of TEs from plant roots to shoots [27].

Interactions between ABA and other phytohormones play a crucial role in regulating plant growth and development. ABA modulates the activity of other hormones by influencing their biosynthesis, thereby affecting their availability and signaling pathways. Under stressful conditions, ABA can act both antagonistically and synergistically with other phytohormones [28]. Furthermore, ABA may indirectly influence SA signaling via its effects on JA signaling, and vice versa [29]. Exogenous application of SA has been shown to enhance ABA production in plants subjected to stress from TEs. Furthermore, the stomatal closure affected by ABA is antagonistically regulated by SA. The interaction between JA and ABA is critical for regulating key processes in plants, such as leaf senescence, stomatal closure and adaptation to various abiotic stresses.

Published studies have predominantly analyzed changes in plant metabolism and stress hormone levels after exogenous application of individual hormones. As the experimental plants are mainly seedlings that have been cultivated for a short period only, the relationship between the regulation of endogenous stress phytohormone levels and TE contents during plant growth and development has not been adequately verified. Although the impacts of individual TEs on plant metabolism have been extensively studied, the effects of combined TEs, which frequently occur in the environment, remain insufficiently understood. To better understand the protective roles of stress phytohormones against combined TE stress, this study compared the effects of TE toxicity on the growth of oat (*Avena sativa* L.) cultivated for four weeks in multi-contaminated soil. To simulate real environmental conditions, the multi-contaminated soil was collected from the Příbram region, an area affected by historical anthropogenic pollution [3]. We hypothesize that soil multi-contamination with TEs significantly disrupts the levels and ratios of stress phytohormones, which are key regulators of plant morphological plasticity. This study aims to investigate whether plant stress levels are influenced by changes in endogenous stress hormone concentrations, their metabolites, and the crosstalk among them. Furthermore, we aim to confirm that plant adaptation to TE-induced stress in multi-contaminated soil results in the different physiological responses in leaves and roots.

## 2. Results

### 2.1. Uptake of Cd, Pb, and Zn and Growth of Oat

**Figure 1:**
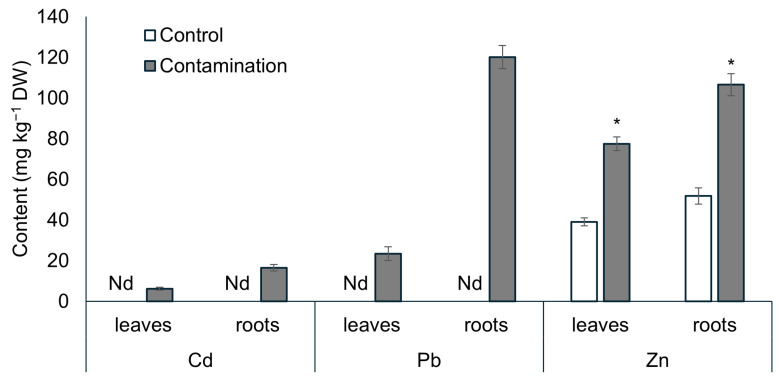
Contents of Cd, Pb, and Zn (mg kg^−1^ dry weight) in the leaves and roots of oat grown in non-polluted soil (Control) and multi-contaminated soil (Contamination). Data are expressed as the four biological replicates’ mean ± standard deviation (SD). Columns marked with asterisks indicate significant differences from the corresponding control values (Kruskal–Wallis ANOVA followed by the Conover–Iman test, *p* < 0.05). Nd—data not determined due to concentration below the detection limit.

The uptake of Cd, Pb, and Zn from the soils was influenced by the varying contents of these TEs in the soil and the ability of oats to accumulate them in the leaves and roots. As shown in Figure 1 and Table S1, oats can accumulate Cd, Pb, and Zn in their biomass when the soil contains elevated levels of these TEs (compared to the control soil, the contents of Cd, Pb, and Zn were 9-, 24-, and 1.5-fold higher, respectively). However, when the soil contents of TEs are low, oats accumulate only Zn in the biomass. In the contaminated treatment, Zn contents in both leaves and roots were twice as high as in the control treatment (Figure 1). The accumulation of Zn in oat leaves and roots strongly correlated with its content in the soil, with correlation coefficients of r = 0.92 ** and r = 0.97 ***, respectively. Under contaminated conditions, all analyzed TEs were predominantly accumulated in the roots. The order of accumulation in the roots was Pb > Zn > Cd, reflecting their contents in the soil, while in the leaves, the order was Zn > Pb > Cd.

**Figure 2:**
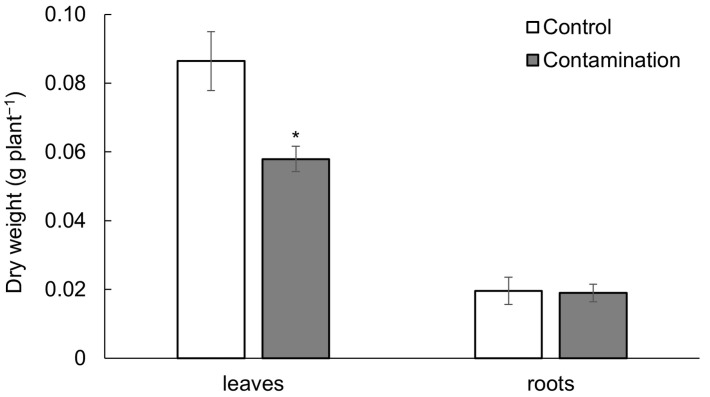
Biomass (dry weight, g plant^−1^) of leaves and roots of oat growing in the non-polluted soil (Control) and multi-contaminated soil (Contamination). Data are expressed as the four biological replicates’ mean ± standard deviation (SD). Columns marked with asterisks indicate significant differences from the corresponding control values (Kruskal–Wallis ANOVA followed by the Conover–Iman test, *p* < 0.05).

Despite the predominant accumulation of the analyzed TEs in oat roots, root dry weight was not significantly affected (Table S1). In contrast, the dry weight of oat leaves decreased by 33% under the contaminated treatment (Figure 2). This effect was supported by a negative correlation between leaf dry weight and the contents of Cd, Pb, and Zn in the soil, with correlation coefficients of r = −0.89 **, r = −0.91 ***, and r = −0.83 *, respectively.

### 2.2. Stress Phytohormones of Oat

Elevated concentrations of Cd, Pb, and Zn in the soil significantly influenced the levels of all bioactive stress phytohormones—JA, SA, and ABA—as well as their metabolites in oat leaves and roots. Among the stress phytohormones, SA and its metabolite salicylic acid (beta)glucoside (SAG) were the most abundant. The second most abundant group comprised jasmonates, particularly the bioactive forms (bJAs). ABA and its metabolites were the least abundant among the detected stress phytohormones in oat leaves and roots.

#### 2.2.1. Jasmonates

**Figure 3:**
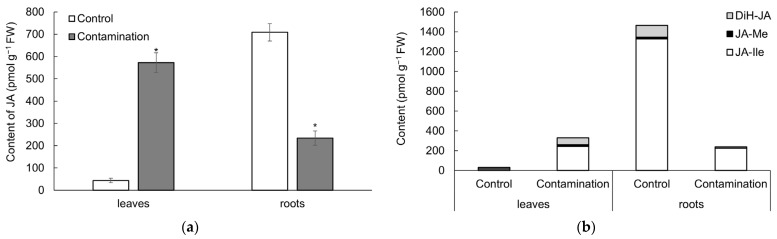
(**a**) Content of jasmonic acid (JA, pmol g^−1^ fresh weight) in leaves and roots of oat growing in the non-polluted soil (Control) and multi-contaminated soil (Contamination). Data are expressed as the four biological replicates’ mean ± standard deviation (SD). Columns marked with asterisks indicate significant differences from the corresponding control values (Kruskal–Wallis ANOVA followed by the Conover–Iman test, *p* < 0.05). (**b**) Content of JA metabolites (pmol g^−1^ fresh weight) in leaves and roots of oat growing in the non-polluted soil (Control) and multi-contaminated soil (Contamination). JA-Ile—JA-isoleucine, JA-Me—jasmonic acid methyl ester, DiH-JA—dihydrojasmonic acid.

The concentrations of endogenous JA and its metabolites—jasmonic acid-isoleucine (JA-Ile), jasmonic acid methyl ester (JA-Me), and dihydrojasmonic acid (DiH-JA)—increased in the leaves of oats exposed to Cd, Pb, and Zn contamination (Figure 3a,b, Table S2). In contrast, a decrease in JA and all its metabolites was observed in the roots. The JA concentration in contaminated leaves was 13-fold higher than that in the control treatment, whereas in the roots it was 3-fold lower (Figure 3a). JA levels in oat leaves positively correlated with soil contents of Cd, Pb, and Zn, with correlation coefficients of r = 0.97 ***, r = 0.99 **, and r = 0.95 ***, respectively. In contrast, JA levels in the roots exhibited strong negative correlations with the same elements: r = −0.98 ***, r = −0.99 ***, and r = −0.96 ***, respectively.

Regarding metabolites, the concentrations of JA-Ile, JA-Me, and DiH-JA in contaminated leaves were 18-, 6-, and 4.8-fold higher than in the control treatment, respectively (Figure 3b). Conversely, in the roots, the levels of JA-Ile, JA-Me, and DiH-JA were 5.9-, 4.9-, and 9.7-fold lower than in the control treatment. A similar pattern was observed for bJAs (the sum of JA and JA-Ile): their content increased 13.9-fold in leaves under contaminated treatment, while in roots it decreased 4.5-fold compared to the control treatment.

The influence of endogenous JA and its metabolites on oat growth suggested strong negative correlations between these metabolites and dry weight of leaves, with correlation coefficients of r = −0.91 ** (JA), r = −0.90 ** (JA-Ile), r = −0.95 *** (JA-Me), and r = −0.91 ** (DiH-JA). In contrast, these metabolites did not correlate with dry weight of roots.

#### 2.2.2. Salicylic Acid

**Figure 4:**
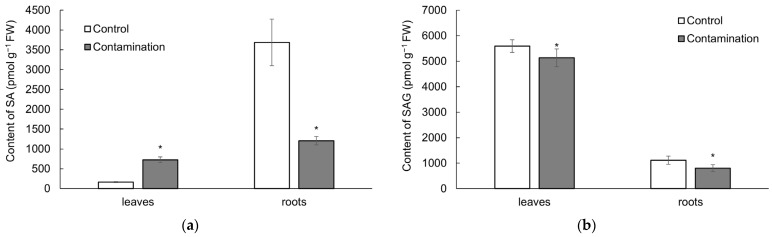
(**a**) Content of salicylic acid (SA, pmol g^−1^ fresh weight) in leaves and roots of oat growing in the non-polluted soil (Control) and multi-contaminated soil (Contamination). Data are expressed as the four biological replicates’ mean ± standard deviation (SD). Columns marked with asterisks indicate significant differences from the corresponding control values (Kruskal–Wallis ANOVA followed by the Conover–Iman test, *p* < 0.05). (**b**) Content of SA metabolite—salicylic acid (beta)glucoside (SAG, pmol g^−1^ fresh weight)—in leaves and roots of oat growing in the non-polluted soil (Control) and multi-contaminated soil (Contamination). Data are expressed as the four biological replicates’ mean ± standard deviation (SD). Columns marked with asterisks indicate significant differences from the corresponding control values (Kruskal–Wallis ANOVA followed by the Conover–Iman test, *p* < 0.05).

Changes in endogenous SA and its metabolite, SAG, in oat leaves and roots are presented in Figure 4a,b and Table S2. SA levels in oat leaves increased under contaminated treatment (4.5-fold higher compared to the control treatment), whereas in roots they decreased (3.1-fold lower compared to the control treatment, Figure 4a). SA concentrations in oat leaves positively correlated with soil contents of Cd, Pb, and Zn, with correlation coefficients of r = 0.97 ***, r = 0.99 ***, and r = 0.93 ***, respectively. In contrast, SA concentrations in oat roots showed significant negative correlations with soil Cd, Pb, and Zn levels: r = −0.93 **, r = −0.95 ***, and r = −0.91 **, respectively. SAG levels decreased under contaminated treatment in both oat leaves and roots, by 8.2- and 1.4-fold, respectively, compared to the control treatment (Figure 4b).

Endogenous SA in oat leaves and roots showed similar influence on growth as observed for JA. SA content negatively correlated with dry weight of leaves (r = −0.91 **), whereas this relationship was not confirmed for roots. SAG levels did not show an effect on growth of both roots and leaves.

#### 2.2.3. Abscisic Acid

**Figure 5:**
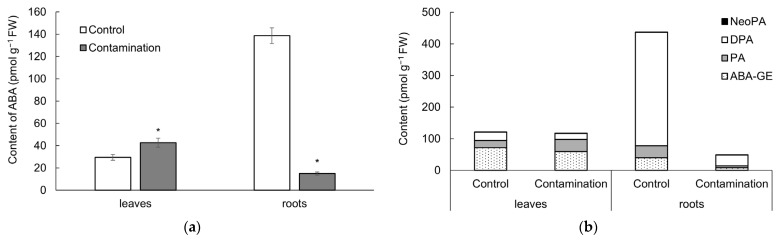
(**a**) Content of abscisic acid (ABA, pmol g^−1^ fresh weight) in leaves and roots of oat growing in the non-polluted soil (Control) and multi-contaminated soil (Contamination). Data are expressed as the four biological replicates’ mean ± standard deviation (SD). Columns marked with asterisks indicate significant differences from the corresponding control values (Kruskal–Wallis ANOVA followed by the Conover–Iman test, *p* < 0.05). (**b**) Content of ABA metabolites (pmol g^−1^ fresh weight) in leaves and roots of oat growing in the non-polluted soil (Control) and multi-contaminated soil (Contamination). ABA-GE—abscisic acid methyl ester, PA—phaseic acid, DPA—dihydrophaseic acid, NeoPA—neophaseic acid.

The levels of endogenous ABA in oat leaves and roots (Figure 5a, Table S2) exhibited responses to the contamination similar to those observed for endogenous JA. Specifically, ABA content in leaves exposed to Cd, Pb, and Zn contamination increased by 1.4-fold compared to the control treatment, whereas in roots it decreased markedly by 9.3-fold. ABA concentrations in leaves showed positive correlations with soil contents of Cd (r = 0.92 **), Pb (r = 0.90 **), and Zn (r = 0.94 ***). Conversely, ABA levels in roots were negatively correlated with these elements: Cd (r = −0.98 ***), Pb (r = −0.99 ***), and Zn (r = −0.95 ***).

Regarding ABA metabolites—abscisic acid methyl ester (ABA-GE), phaseic acid (PA), dihydrophaseic acid (DPA), and neophaseic acid (NeoPA)—the responses were variable (Figure 5b, Table S2). Although the total content of ABA metabolites in leaves and roots did not significantly differ under Cd, Pb, and Zn contamination, individual metabolites showed distinct changes. Levels of ABA-GE decreased by 0.8-fold in leaves and 4.7-fold in roots compared to the control treatment. A similar pattern was observed for DPA, with decreases of 0.7-fold in leaves and 10.7-fold in roots. In contrast, PA and NeoPA increased in leaves by 1.7-fold and 1.4-fold, respectively, but decreased in roots by 6.1-fold and 4.4-fold, respectively, relative to the control treatment.

Possible influence of endogenous ABA and its metabolites (except of NeoPA) on oat growth suggested correlation between individual phytohormone and dry weight. Both negative and positive correlations were observed for leaves, with correlation coefficients of r = −0.74 * (ABA), r = 0.86 * (ABA-GE), r = −0.89 ** (PA), and r = 0.77 * (DPA). In contrast, in oat roots, correlations were not observed.

#### 2.2.4. Relationships Between Stress Phytohormones

**Figure 6:**
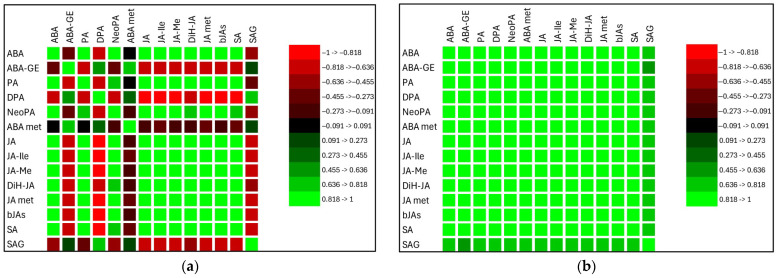
(**a**) Correlation matrix between stress phytohormones in oat leaves (*p* < 0.05). (**b**) Correlation matrix between stress phytohormones in oat roots (*p* < 0.05). ABA—abscisic acid, ABA-GE—abscisic acid methyl ester, ABA met—sum of abscisic acid metabolites, PA—phaseic acid, DPA—dihydrophaseic acid, NeoPA—neophaseic acid, JA—jasmonic acid, JA-Ile—JA-isoleucine, JA-Me—jasmonic acid methyl ester, DiH-JA—dihydrojasmonic acid, JA met—sum of jasmonic acid metabolites, bJAs—sum of bioactive forms of jasmonic acid, SA—salicylic acid, SAG—salicylic acid (beta)glucoside.

Pearson’s correlation coefficient was used to identify significant relationships among stress phytohormones. Data from both treatments were evaluated together. Positive correlations among endogenous phytohormones were observed in roots, whereas the relationships in leaves were more variable (Figure 6a,b). Results for oat roots were significant, except for SA and SAG (Table S3), and suggest a synergistic interaction among stress phytohormones and their metabolites. Similarly, the majority of correlation coefficients were significant in oat leaves (Table S3). Among these phytohormones, ABA showed strong correlations with JA in both roots (r = 0.99 ***) and leaves (r = 0.94 **), and with SA in roots (r = 0.95 ***) and leaves (r = 0.88 *). A significant correlation between ABA and the sum of its metabolites was observed only in oat roots (r = 0.99 ***). For jasmonates, JA and its metabolites were strongly correlated in both roots (r = 0.98 ***) and leaves (r = 0.99 ***). In contrast, a significant correlation between SA and its metabolite SAG was found only in oat leaves (r = −0.71 *).

## 3. Discussion

### 3.1. Cd, Pb, and Zn Accumulation and Its Influence on the Growth of Oat

The content of TEs in plant biomass depends on their concentration in the soil, and their availability to plants can be reduced due to antagonistic interactions among elements. For instance, Orroñoa et al. [30] reported reduced Pb availability in the presence of five heavy metals—Cd, Zn, Cr, Cu, and Ni. Our findings align with this observation, confirming higher TE contents in the contaminated treatment. All studied TEs in the contaminated treatment accumulated predominantly in the roots, in the order Pb > Zn > Cd. It is well established that Pb tends to accumulate in plant roots [31]. Greater root accumulation of Pb compared to leaves has been documented in oats [32,33]. Dogan et al. [34] suggested that Pb accumulation in root cells is due to the barrier effect of Casparian strips.

Cd accumulation in roots and leaves (roots > leaves) increases with rising Cd concentrations in soil. Cd toxicity inhibits the activity of chlorophyll-ester reductase, thereby reducing chlorophyll synthesis and leading to the disruption of the plant photosynthetic system structure [35]. Under conditions of excessive evapotranspiration, Cd permeates the cytosol through Ca channels in the plasmalemma, impairing cellular water status [36]. As a mobile element within the plant, Cd may disrupt the nutritional status of various plant parts, primarily by interfering with the coordination between C and N metabolism [37].

The translocation of TEs from roots to leaves followed the sequence Zn > Pb >> Cd, indicating differential uptake and mobility. Zinc is an essential micronutrient for plants. Zemanová et al. [5] demonstrated that Zn concentrations up to 300 mg Zn kg^−1^ dry weight positively affect the metabolic activity of spinach. Marschner [38] noted that optimal Zn levels in plant leaves range from 30 to 200 mg Zn kg^−1^ dry weight. In our study, Zn levels in oat leaves did not reach toxic thresholds. The elevated Zn concentration likely supported the metabolic functions of oats and may have reduced Pb availability to the plant roots.

Plant adaptation to TE-induced stress typically results in reduced growth and biomass accumulation. Rehman et al. [39] reported a 25–30% yield reduction in wheat under Pb stress. Similarly, Pb toxicity led to a reduction in oat biomass by 67% in leaves and 74% in roots [32]. Howladar et al. [40] observed a 77% decline in wheat roots biomass under Cd toxicity. Furthermore, combined contamination with multiple TEs (As, Cd, Pb, Zn) reduced leaf lettuce biomass by 48% [5]. Common visible symptoms of TE toxicity include chlorosis, browning, and necrosis of leaves [4,41,42,43]. Our pot experiment mirrored these findings, showing a significant reduction in leaf biomass under contamination compared to the control.

### 3.2. Stress Phytohormones of Oat Under Cd, Pb, and Zn Influence

Phytohormone metabolism modulates the pool of bioactive molecules, thereby influencing plant growth and development under both stress and non-stress conditions. Plant stress is characterized by altered phytohormone levels and ratios, which play a pivotal role in mediating morphological plasticity [4,44,45].

In control plants, JA was predominantly concentrated in the roots, whereas contaminated plants exhibited increased JA levels in leaves. Our results indicate that TE contamination stimulates the accumulation of endogenous JA and its metabolites in oat leaves. Numerous studies confirm that JA levels rise under TE stress, accompanied by the upregulation of JA-biosynthetic or JA-responsive genes [20,21,46,47]. Exposure of plants to Cd stress rapidly activates the JA signaling pathway, which positively regulates the Cd response by repressing the transcriptional levels of genes involved in Cd uptake and root-to-shoot translocation [48]. JA has been shown to enhance Cd resistance in rice. A reduced capacity of root cell wall hemicelluloses to sequester Cd, leading to decreased Cd uptake into root cells, has also been observed [49].

JA is predominantly involved in plant stress responses and can inhibit growth [21]. The observed elevation in JA levels, along with leaf growth inhibition, supports this. Cd-induced JA plays a protective role under Cd stress by suppressing the expression of non-discriminative transporter genes involved in Cd uptake and translocation. This suppression leads to a reduction in transporter abundance, thereby decreasing Cd concentrations in both roots and shoots and ultimately mitigating Cd toxicity [50].

Jasmonates enhance ROS scavenging, increase antioxidant enzyme activity, promote chelation, and modulate ion transport [18]. JA is further regulated through modification processes, including methylation by JA methyl transferase to produce JA-Me and conjugation with amino acids by JA conjugate synthase to form JA-Ile [51]. The highest contents of JA metabolites were found in the roots of control plants. Similarly, leaves of contaminated plants exhibited increased JA metabolites levels. The most bioactive form, JA-Ile, an amide conjugate with an amino acid [52], was the predominant JA metabolite in oat. In ferns (*Pteris* sp.) growing in As contaminated soil, JA-Ile levels also increased in fronds during growth [44]. According to Wasternack and Strnad [53], under normal growth conditions, genes involved in JA biosynthesis remain inactivated, resulting in low levels of JA-Ile in the cytoplasm. Under stress, JA undergoes epimerization to form JA-Ile, which accumulates in the cytoplasm of stressed leaves. At high cytosolic concentrations, JA-Ile is transported through the nuclear membrane into the nucleus via JA transporter proteins such as JAT1 [54]. The observed increase in JA levels in plants grown on contaminated soil suggests enhanced membrane degradation processes, supporting the role of jasmonates in promoting leaf senescence under stress conditions.

Endogenous SA levels displayed a similar pattern to JA, with increased SA in leaves and decreased SA in roots under contamination. According to Verma et al. [55], both antagonistic and synergistic interactions between these two stress phytohormones have been demonstrated. Our results suggest a synergistic interaction between JA and SA in oat leaves and roots under TE stress. SA and its metabolites elicit an acclimation response and enhance resistance to TE stress by modulating metabolic processes—primarily through the induction of antioxidant capacity and the synthesis of non-protein thiols [56]. Freeman et al. [57] reported increased accumulation of endogenous SA and its metabolites in several *Thlaspi* species exhibiting Ni/Zn hyperaccumulation. According to the authors, the enhanced tolerance of certain plants to TEs is mediated by glutathione and signaled by constitutively elevated SA levels. Exogenous SA is known to improve TE stress tolerance through activation of the antioxidant system, improved glutathione metabolism, and upregulation of defense-related genes [16,58,59]. For example, SA decreased the oxidative burst of Pb stress in *Phaseolus vulgaris* [60]. The study by Pál et al. [61] demonstrated that SA inhibits phytochelatin synthase activity to preserve effective glutathione levels in the cytosol, thereby enabling its efficient function as an antioxidant. For example, Zn-stressed plants showed a 54% decline in SA levels compared to controls [16]. A significant SA reduction was also noted in lupine roots grown in multi-contaminated soil [43]. Zemanová et al. [44] recorded declining SA levels in *Pteris* sp. roots over 185 days of growth, which our results corroborate for oats. Emamverdian et al. [12] suggested that low SA levels can enhance stress tolerance by boosting antioxidant enzyme activity and reducing oxidative damage. Conversely, Szalai and Janda [62] observed that excessive SA can cause tissue injury via increased ROS generation in photosynthetic tissues [63]. Our data support this, with elevated SA leaves correlating with reduced leaf growth. The higher SA levels in leaves compared to roots suggest the presence of rapid upward transport of SA to aerial plant organs, followed by its conversion into SA glucoside, predominantly within the leaf tissues [56]. In plants, SA exists in both an active free form and inactive storage form (SA glucoside and SA glucose ester) [64]. The active form, SAG, decreased in both leaves and roots under contaminated treatment. Li et al. [65] reported declines in both SA and SAG in tomato under boron deficiency combined with manganese and copper excess. Similarly, reduced SAG in soybean roots under abiotic stress correlated with elevated L-tryptophan, a precursor of auxin synthesis [66]. Thus, alterations in JA and SA levels contribute to reduced leaf and root length under TE stress. Consistent with the cited conclusion, we propose that under multi-TE stress conditions, SA fulfills a multifaceted role—functioning as a signaling molecule, a regulator of oxidative stress, and potentially as a chelator of TEs involved in detoxification mechanisms.

Abscisic acid plays a central role in numerous biological processes and responses to abiotic stresses [17]. Elevated ABA levels have been observed in various plants under TE exposure [67]. As a growth regulator, ABA enhances stress resistance: low ABA levels stimulate root elongation, while high levels inhibit it [28]. This contradiction can lead to increased TE accumulation in roots due to reduced translocation to shoots. In our study, ABA levels significantly declined in oat roots under contamination, whereas leaf ABA levels increased. The increase in endogenous ABA levels observed in leaves under the contamination treatment, compared to the control, aligns with the physiological processes associated with leaf senescence. A key factor contributing to the onset of senescence is chlorophyll degradation [68]. Furthermore, changes in ABA levels exhibited a pattern similar to that of JA in response to contamination, which, together with correlation, suggest a synergetic interaction in oats. ABA is capable of long-distance transport from roots to leaves and from xylem to guard cells, contributing to stomatal regulation and water balance under TE stress [12]. Moreover, ABA plays a mediating role in N utilization under Cd stress, notably by significantly suppressing N uptake and translocation of NO_3_-N to the shoots. These findings indicate that ABA negatively regulates NO_3_^−^-N uptake by inhibiting key transporters [37].

The regulation of ABA in plants is governed by both its biosynthetic and catabolic pathways [69]. ABA catabolism proceeds through hydroxylation and conjugation reactions. Glycosylation involves the conjugation of ABA with glucose to form ABA-GE, the inactive compound. This process is reversible [70,71]. The conjugation/deconjugation cycle enables plants to adapt to environment conditions through ABA-mediated responses by allowing rapid activation and inactivation of ABA [72]. Under stress conditions, ABA-glucose ester (ABA-GE) is converted into active ABA by β-glucosidases [73]. This observation is supported by our results, which show a decrease in ABA-GE levels in the roots under the contamination treatment. ABA-GE itself lacks biological activity and is considered a transportable form of ABA, capable of being translocated from the cytoplasm to the vacuole and endoplasmic reticulum, where it is stored [73].

Oxidative degradation of ABA, catalyzed by ABA-8′-hydroxylase, leads to the formation of 8′-hydroxy ABA or 9-hydroxy ABA, which are subsequently rearranged into PA and then reduced to DPA. This catabolic pathway is regarded as a key regulatory mechanism that attenuates ABA signaling. The resulting metabolites—PA, DPA, and its glucosylated form DPAG (4-β-glucoside)—are generally considered inactive. However, despite being a catabolite, PA also functions as a signaling molecule in plant physiology and environmental adaptation, acting alongside ABA in higher plants. PA exhibits ABA-like hormonal activity in certain species, including the regulation of stomatal closure [74]. In our study, we observed elevated PA levels in leaves under the contamination treatment compared to the control treatment. Among ABA metabolites in roots, DPA represents the highest proportion, and its level was significantly reduced in oat roots subjected to the contaminated treatment. This finding suggests that PA signaling can be effectively downregulated through its metabolic conversion to DPA, indicating that ABA inactivation in oat roots occurs primarily via DPA formation. Notably, low levels of bioactive ABA are known to promote root elongation. Zemanová et al. [44] reported comparable findings in the non-hyperaccumulating fern *Pteris straminea*. No significant treatment differences were observed in leaf metabolite content. In leaves, ABA-GE was the predominant metabolite, reflecting ABA inactivation via glycosylation. Under stress, increased ABA-GE levels in xylem sap suggest active long-distance transport of ABA [28].

## 4. Materials and Methods

### 4.1. Plant Growing Experiment

#### 4.1.1. Soils

Two soils from different localities were used in the pot experiment. The first soil, chernozem haplic, was collected from Prague-Suchdol, Czech Republic (50°8′8″ N, 14°22′43″ E) and used as the control. According to Czech standards for TE content in soil (0.5 mg kg^−1^ for Cd, 60 mg kg^−1^ for Pb, and 120 mg kg^−1^ for Zn), this soil was classified as non-polluted [75]. Its basic parameters were as follows: pH_H2O_—7.5, cation exchange capacity—230.1 ± 5.0 mmol_(+)_ kg^−1^, total carbon—2.0 ± 0.1%, pseudo-total content of Cd—0.4 ± 0.01 mg kg^−1^, pseudo-total content of Pb—41.4 ± 3.2 mg kg^−1^, and pseudo-total content of Zn—92.6 ± 2.9 mg kg^−1^. The second soil, a cambisol haplic, was collected from the locality of Podlesí near the city of Příbram in the Czech Republic (49°42′24″ N, 13°58′32″ E) and was used as the contaminated soil. This soil was classified as polluted due to historical mining and smelting activities [3,76]. Its basic parameters were as follows: pH_H2O_—6.0, cation exchange capacity—165.8 ± 15.1 mmol_(+)_ kg^−1^, total carbon—2.4 ± 0.1%, pseudo-total content of Cd—3.9 ± 0.6 mg kg^−1^, pseudo-total content of Pb—1003.2 ± 95.7 mg kg^−1^, and pseudo-total content of Zn—142.1 ± 13.0 mg kg^−1^.

The experiment was conducted in a greenhouse with four replications of each soil type arranged in a randomized design. Each pot in the control group was filled with 2.5 kg of soil and 2.5 kg of washed silica sand (particle size 0.4 mm) to lighten the soil texture. Each pot in the contamination group was filled with 5 kg of soil. Nutrients were added to each pot, consisting of 0.5 g N, 0.16 g P, and 0.4 g K, which were applied as NH_4_NO_3_ and K_2_HPO_4_ solutions per pot.

#### 4.1.2. Plants

Oat (*Avena sativa* L.) seeds, purchased from AROS-osiva s.r.o. (Prague, Czech Republic), were sown in individual pots at a rate of 25 seeds per pot. The plants were cultivated under semi-controlled conditions, including natural light, a 16 h photoperiod, a daytime temperature of 22 °C, a nighttime temperature of 18 °C, and a relative humidity of ~60%. After 4 weeks, the plants were harvested and separated into leaves and roots. The biomass was rinsed with demineralized water, blotted dry, weighed, and divided for further analysis. One portion was immediately frozen in liquid nitrogen and stored at –80 °C for phytohormone analysis. The other portion was oven-dried at 40 °C until it reached a constant weight, after which it was homogenized for TE analysis.

### 4.2. Chemical Analysis

#### 4.2.1. Determination of Toxic Elements

The contents of TEs were measured as previously described [5,77]. Dry biomass was digested under low-pressure conditions using a 4:1 (*v*/*v*) mixture of HNO_3_ and H_2_O_2_ (10 mL; Ethos 1, MLS GmbH, Leutkirch im Allgäu, Germany). TEs were determined in the solutions using an Agilent 720 inductively coupled plasma optical emission spectrometer (ICP-OES; Agilent Technologies Inc., Santa Clara, CA, USA).

#### 4.2.2. Determination of Phytohormones

The stress phytohormones were extracted from fresh biomass as previously described [78]. The contents were analyzed using an LC/MS system consisting of a UHPLC 1290 Infinity II (Agilent Technologies Inc., Santa Clara, CA, USA) coupled with a 6495 Triple Quadrupole Mass Spectrometer (Agilent Technologies Inc.). The system operated in MRM mode, with the isotope dilution method for quantification. Data acquisition and processing were performed using Mass Hunter software B.08 (Agilent Technologies Inc., Santa Clara, CA, USA).

### 4.3. Statistical Analysis

Statistical analyses were conducted using XLStat version 2023.1.3 software (Lumivero, Burlington, MA, USA). Results are expressed as mean values with standard deviations (SD) for four biological replicates per soil. Data normality was tested using the Shapiro–Wilks test. Since the data were non-normally distributed, the Kruskal–Wallis ANOVA was employed, followed by the Conover–Iman procedure. Pearson’s linear correlation analysis was also performed to demonstrate the relationship between variables. Calculated relations were significant at *p* < 0.05 (*), *p* < 0.01 (**), and *p* < 0.001 (***).

## 5. Conclusions

Based on the findings of this study, it can be concluded that plant adaptation to stress induced by soil multi-contamination with TEs elicits differential responses in leaves and roots. TE-related stress was evidenced by significant alterations in the levels of all bioactive stress phytohormones (JA, SA, and ABA), as well as their metabolites, in both oat leaves and roots. For all three phytohormones, the highest concentrations were observed in the roots of unstressed plants, with marked reduction in the roots of plants subjected to TE contamination. In contrast, leaf responses exhibited the opposite trend. Lower JA and SA levels in roots may enhance plant tolerance and strengthen defense mechanisms by inducing antioxidant enzyme activities, thereby reducing oxidative stress. Low ABA levels are known to stimulate root elongation. The distribution pattern of JA metabolites paralleled the changes in JA levels. The bioactive JA form, JA-Ile, represented the largest proportion of identified JA metabolites in oat. Similarly, the active form of SA, SAG, decreased in both leaves and roots under TE-induced stress. Among ABA metabolites detected in the roots, DPA was the most abundant; its significant reduction in contaminated treatment suggests that ABA inactivation proceeds primarily through the DPA pathway.

## Data Availability

The data presented in this study are available in the article.

## Supplementary Materials

The following supporting information can be downloaded at: https://www.mdpi.com/article/10.3390/plants14162554/s1, Table S1: Accumulation of Cd, Pb, Zn and dry biomass of oat leaves and roots; Table S2: Stress phytohormones and their metabolites in the leaves and roots of oat; Table S3: Correlation coefficients of phytohormones in the leaves and roots of oat.

## References

[1] Hou D., Jia X., Wang L., McGrath S.P., Zhu Y.-H., Hu Q., Zhao F.-J., Bank M.S., O’Connor D., Nriagu J. (2025). Global soil pollution by toxic metals threatens agriculture and human health. Science.

[2] Wang H., Yu S., Sun L., Wang Y., Wu H., Wang X. (2025). Pollution assessment and health risk of metals in surface soil near a Pb–Zn mine, northeast China. Front. Environ. Sci..

[3] Vaněk A., Ettler V., Grygar T., Borůvka L., Šebek O., Drábek O. (2008). Combined chemical and mineralogical evidence for heavy metal binding in mining- and smelting-affected alluvial soils. Pedosphere.

[4] Hajam A.H., Ali M.S., Singh S.K., Bashri G. (2024). Understanding cytokinin: Biosynthesis, signal transduction, growth regulation, and phytohormonal crosstalk under heavy metal stress. Environ. Exp. Bot..

[5] Zemanová V., Lhotská M., Novák M., Hnilička F., Popov M., Pavlíková D. (2024). Multicontamination toxicity evaluation in the model plant *Lactuca sativa* L.. Plants.

[6] Pavlíková D., Zemanová V., Pavlík M., Lhotská M., Kubeš J., Novák M., Dobrev P.I., Motyka V. (2025). Phytohormone and amino acid changes in cherry radish as metabolic adaptive response to arsenic single and multi-contamination. Biomolecules.

[7] Liu J., He H., Vitali M., Visentin I., Charnikhova T., Haider I., Schubert A., Ruyter-Spira C., Bouwmeester H.J., Lovisolo C. (2015). Osmotic stress represses strigolactone biosynthesis in *Lotus japonicus* roots: Exploring the interaction between strigolactones and ABA under abiotic stress. Planta.

[8] Ronzana M., Piacentinia D., Fattorinia L., Della Roverea F., Eicheb E., Riemannc M., Altamuraa M.M., Falasca G. (2018). Cadmium and arsenic affect root development in *Oryza sativa* L. negatively interacting with auxin. Environ. Exp. Bot..

[9] Jamla M., Khare T., Joshi S., Patil S., Penna S., Kumar V. (2021). Omics approaches for understanding HMs responses and tolerance in plants. Curr. Plant Biol..

[10] Sytar O., Ghosh S., Malinska H., Zivcak M., Brestic M. (2021). Physiological and molecular mechanisms of metal accumulation in hyperaccumulator plants. Physiol. Plant..

[11] Urano K., Maruyama K., Jikumaru Y., Kamiya Y., Yamaguchi-Shinozaki K., Shinozaki K. (2017). Analysis of plant hormone profiles in response to moderate dehydration stress. Plant J..

[12] Emamverdian A., Ding Y., Mokhberdoran F. (2020). The role of salicylic acid and gibberellin signaling in plant responses to abiotic stress with an emphasis on heavy metals. Plant Signal. Behav..

[13] Sharma A., Sidhu G.P.S., Araniti F., Bali A.S., Shahzad B., Tripathi D.K., Brestic M., Skalicky M., Landi M. (2020). The Role of Salicylic Acid in Plants Exposed to Heavy Metals. Molecules.

[14] Ali E., Hussain S., Jalal F., Khan M.A., Imtiaz M., Said F., Ismail M., Khan S., Ali H.M., Hatamleh A.A. (2023). Salicylic acid-mitigates abiotic stress tolerance via altering defense mechanisms in *Brassica napus* (L.). Front. Plant Sci..

[15] Torun H., Cetin B., Stojnic S., Petrík P. (2024). Salicylic acid alleviates the effects of cadmium and drought stress by regulating water status, ions, and antioxidant defense in *Pterocarya fraxinifolia*. Front. Plant Sci..

[16] Li Q., Guan C., Zhao Y., Duan X., Yang Z., Zhu J. (2023). Salicylic acid alleviates Zn-induced inhibition of growth via enhancing antioxidant system and glutathione metabolism in alfalfa. Ecotoxicol. Environ. Saf..

[17] Rahman S.U., Li Y., Hussain S., Hussain B., Khan W.-U.-D., Riaz L., Ashraf M.N., Khaliq M.A., Du Z., Cheng H. (2023). Role of phytohormones in heavy metal tolerance in plants: A review. Ecol. Indic..

[18] Bilal S., Saad Jan S., Shahid M., Asaf S., Khan A.L., Lubna, Al-Rawahi A., Lee I.-J., AL-Harrasi A. (2023). Novel insights into exogenous phytohormones: Central regulators in the modulation of physiological, biochemical, and molecular responses in rice under metal(loid) stress. Metabolites.

[19] Ndecky S., Malherbe L., Villette C., Chalvon V., Meusnier I., Beltran-Valencia D., Baumberger N., Riemann M., Kroj T., Champion A. (2025). Rice JASMONIC ACID OXIDASES control resting jasmonate metabolism to promote growth and repress basal immune responses. Plant Physiol..

[20] Per T.S., Khan M.I.R., Anjum N.A., Masood A., Hussain S.J., Khan N.A. (2018). Jasmonates in plants under abiotic stresses: Crosstalk with other phytohormones matters. Environ. Exp. Bot..

[21] Chen X., Jiang W., Tong T., Chen G., Zeng F., Jang S., Gao W., Li Z., Mak M., Deng F. (2021). Molecular interaction and evolution of jasmonate signaling with transport and detoxification of heavy metals and metalloids in plants. Front. Plant Sci..

[22] Hu B., Deng F., Chen G., Chen X., Gao W., Long L., Xia L., Chen Z.-H. (2020). Evolution of Abscisic Acid Signaling for Stress Responses to Toxic Metals and Metalloids. Front. Plant Sci..

[23] Zhao Y., Wang J., Huang W., Zhang D., Wu J., Li B., Li M., Liu L., Yan M. (2023). Abscisic-acid-regulated responses to alleviate cadmium toxicity in plants. Plants.

[24] Leng Y., Li Y., Ma Y.-H., He L.-F., Li S.-W. (2020). Abscisic acid modulates differential physiological and biochemical responses of roots, stems, and leaves in mung bean seedlings to cadmium stress. Environ. Sci. Pollut. Res..

[25] Cobbett C., Goldsbrough P. (2002). Phytochelatins and metallothioneins: Roles in heavy metal detoxification and homeostasis. Annu. Rev. Plant Biol..

[26] Kim T.-H., Böhmer M., Hu H., Nishimura N., Schroeder J.I. (2010). Guard cell signal transduction network: Advances in understanding abscisic acid, CO_2_, and Ca^2+^ signaling. Annu. Rev. Plant Biol..

[27] Kumar S., Shah S.H., Vimala Y., Jatav H.S., Ahmad P., Chen Y., Siddique K.H.M. (2022). Abscisic acid: Metabolism, transport, crosstalk with other plant growth regulators, and its role in heavy metal stress mitigation. Front. Plant Sci..

[28] Singh A., Roychoudhury A. (2023). Abscisic acid in plants under abiotic stress: Crosstalk with major phytohormones. Plant Cell Rep..

[29] Wani A.B., Chadar H., Wani A.H., Singh S., Upadhyay N. (2017). Salicylic acid to decrease plant stress. Environ. Chem. Lett..

[30] Orroño D.I., Schindler V., Lavado R.S. (2012). Heavy metal availability in *Pelargonium hortorum* rhizosphere: Interactions, uptake and plant accumulation. J. Plant Nutr..

[31] Fahr M., Laplaze L., Bendaou N., Hocher V., El Mzibri M., Bogusz D., Smouni A. (2013). Effect of lead on root growth. Front. Plant Sci..

[32] Jatav P.K., Verma R., Kothari S.L., Jain R., Kachhwaha S. (2021). Relative morpho-physiological responses of millets and oats against lead toxicity. Environ. Exp. Bot..

[33] Piršelová B., Galuščáková Ľ., Lengyelová L., Kubová V., Matúšová R., Bojnanská K., Havrlentová M. (2024). Phytoremediation potential of oat (*Avena sativa* L.) in soils contaminated with cadmium. Agron. Res..

[34] Dogan M., Karatas M., Aasim M. (2018). Cadmium and lead bioaccumulation potentials of an aquatic macrophyte *Ceratophyllum demersum* L.: A laboratory study. Ecotoxicol. Environ. Saf..

[35] Gill S.S., Khan N.A., Tuteja N. (2012). Cadmium at high dose perturbs growth, photosynthesis and nitrogen metabolism while at low dose it up regulates sulfur assimilation and antioxidant machinery in garden cress (*Lepidium sativum* L.). Plant Sci..

[36] Perfus-Barbeoch L., Leonhardt N., Vavasseur A., Forestier C. (2002). Heavy metal toxicity: Cadmium permeates through calcium channels and disturbs the plant water status. Plant J..

[37] Xu Z.J., Qu J.Y., Zhao M., Zhao J., Wang W.C., Huang J.J., Peng J.S., Xiao Y.H., Han Y.L., Peng Y. (2025). Nitrogen utilization in response to cadmium and abscisic acid in rice. Plant Soil.

[38] Marschner P. (2012). Marschner’s Mineral Nutrition of Higher Plants.

[39] Rehman M.Z.U., Rizwan M., Ali S., Sabir M., Sohail M.I. (2017). Contrasting effects of organic and inorganic amendments on reducing lead toxicity in wheat. Bull. Environ. Contam. Toxicol..

[40] Howladar S.M., Al-Robai S.A., Al-Zahrani F.S., Howladar M.M., Aldhebiani A.Y. (2018). Silicon and its application method effects on modulation of cadmium stress responses in *Triticum aestivum* (L.) through improving the antioxidative defense system and polyamine gene expression. Ecotoxicol. Environ. Saf..

[41] Chaturvedi R., Favas P.J.C., Pratas J., Varun M., Paul M.S. (2019). Metal(loid) induced toxicity and defense mechanisms in *Spinacia oleracea* L. Ecological hazard and prospects for phytoremediation. Ecotox. Environ. Saf..

[42] Rashid A., Schutte B.J., Ulery A., Deyholos M.K., Sanogo S., Lehnhoff E.A., Beck L. (2023). Heavy metal contamination in agricultural soil: Environmental pollutants affecting crop health. Agronomy.

[43] Novák M., Zemanová V., Černý J., Pavlíková D. (2024). Roots of *Lupinus angustifolius* L. and enzyme activities in soil contaminated by toxic elements. Plant Soil Environ..

[44] Zemanová V., Pavlíková D., Dobrev P.I., Motyka V., Pavlík M. (2019). Endogenous phytohormone profiles in *Pteris* fern species differing in arsenic accumulating ability. Environ. Exp. Bot..

[45] Zheng Y., Wang X., Cui X., Wang K., Wang Y., He Y. (2023). Phytohormones regulate the abiotic stress: An overview of physiological, biochemical, and molecular responses in horticultural crops. Front. Plant Sci..

[46] Dar T.A., Moinuddin Khan M.M.A., Hakeem K.R., Jaleel H. (2015). Jasmonates counter plant stress: A review. Environ. Exp. Bot..

[47] Kim H., Seomun S., Yoon Y., Jang G. (2021). Jasmonic acid in plant abiotic stress tolerance and interaction with abscisic acid. Agronomy.

[48] Zhang H., Liu Z., Li X., Liu X., Fang L., Zeng R., Wang Q., Song Y., Chen D. (2025). Jasmonic acid enhances rice cadmium tolerance by suppressing cadmium uptake and translocation. Plants.

[49] Yang J.B., Wang H.Y., Huang J., Shan C.J., Yan J., Zhong C.W., Hu D., Zhang Q., Shen R.F., Zhu X.F. (2025). Jasmonic acid improves cadmium tolerance in rice (*Oryza sativa*) by reducing the production of nitric oxide. Ecotox. Environ. Saf..

[50] Lei G.J., Sun L., Sun Y., Zhu X.F., Li G.X., Zheng S.J. (2020). Jasmonic acid alleviates cadmium toxicity in *Arabidopsis* via suppression of cadmium uptake and translocation. J. Integr. Plant Biol..

[51] Han G.Z. (2017). Evolution of jasmonate biosynthesis and signaling mechanisms. J. Exp. Bot..

[52] Li N., Han X., Feng D., Yuan D., Huang L.H. (2019). Signaling crosstalk between salicylic acid and ethylene/jasmonate in plant defense: Do we understand what they are whispering?. Int. J. Mol. Sci..

[53] Wasternack C., Strnad M. (2016). Jasmonate signaling in plant stress responses and development—Active and inactive compounds. N. Biotechnol..

[54] Ruan J., Zhou Y., Zhou M., Yan J., Khurshid M., Weng W., Cheng J., Zhang K. (2019). Jasmonic acid signaling pathway in plants. Int. J. Mol. Sci..

[55] Verma V., Ravindran P., Kumar P.P. (2016). Plant hormone-mediated regulation of stress responses. BMC Plant Biol..

[56] Drzewiecka K., Mleczek M. (2017). Salicylic acid accumulation as a result of Cu, Zn, Cd and Pb interactions in common reed (*Phragmites australis*) growing in natural ecosystems. Acta Physiol. Plant..

[57] Freeman J.L., Persans M.W., Nieman K., Albrecht C., Peer W., Pickering I.J., Salt D.E. (2004). Increased glutathione biosynthesis plays a role in nickel tolerance in *Thlaspi* nickel hyperaccumulators. Plant Cell.

[58] Wang H., Feng T., Peng X., Yan M., Tang X. (2009). Up-regulation of chloroplastic antioxidant capacity is involved in alleviation of nickel toxicity of *Zea mays* L. by exogenous salicylic acid. Ecotoxicol. Environ. Saf..

[59] Agami R.A., Mohamed G.F. (2013). Exogenous treatment with indole-3-acetic acid and salicylic acid alleviates cadmium toxicity in wheat seedlings. Ecotox. Environ. Saf..

[60] Khalil R., Haroun S., Bassyoini F., Nagah A., Yusuf M. (2021). Salicylic acid in combination with kinetin or calcium ameliorates HMs stress in *Phaseolus vulgaris* plant. J. Agric. Food Res..

[61] Pál M., Szalai G., Horváth E., Janda T., Páldi E. (2002). Effect of salicylic acid during heavy metal stress. Acta Biol. Szeged..

[62] Szalai G., Janda T. (2007). Induction of abiotic stress tolerance by salicylic acid signaling. J. Plant Growth Regul..

[63] Jumali S.S., Said I.M., Ismail I., Zainal Z. (2011). Genes induced by high concentration of salicylic acid in *Mitragyna speciosa*. Aust. J. Crop. Sci..

[64] Mishra A.K., Baek K.-H. (2021). Salicylic acid biosynthesis and metabolism: A divergent pathway for plants and bacteria. Biomolecules.

[65] Li J., Fan H., Song Q., Jing L., Yu H., Li R., Zhang P., Liu F., Li W., Sun L. (2023). Physiological and molecular bases of the boron deficiency response in tomatoes. Hortic. Res..

[66] Qiu G., Han Z., Wang Q., Wang T., Sun Z., Yu Y., Han X., Yu H. (2023). Toxicity effects of nanoplastics on soybean (*Glycine max* L.): Mechanisms and transcriptomic analysis. Chemosphere.

[67] Saini S., Kaur N., Pati P.K. (2021). Phytohormones: Key players in the modulation of heavy metal stress tolerance in plants. Ecotoxic. Environ. Saf..

[68] Lim J., Lim C.W., Lee S.C. (2022). Core components of abscisic acid signaling and their post-translational modification. Front. Plant Sci..

[69] Long H., Zheng Z., Zhang Y., Xing P., Wan X., Zheng Y., Li L. (2019). An abscisic acid (ABA) homeostasis regulated by its production, catabolism and transport in peanut leaves in response to drought stress. PLoS ONE.

[70] Nambara E., Marion-Poll A. (2005). Abscisic acid biosynthesis and catabolism. Annu. Rev. Plant Biol..

[71] Vishwakarma K., Upadhyay N., Kumar N., Yadav G., Singh J., Mishra R.K., Kumar V., Verma R., Upadhyay R.G., Pandey M. (2017). Abscisic acid sgnaling and abiotic stress tolerance in plants. Front. Plant Sci..

[72] Chen K., Li G.J., Bressan R.A., Song C.P., Zhu J.K., Zhao Y. (2020). Abscisic acid dynamics, signaling, and functions in plants. J. Integr. Plant Biol..

[73] Liu Y., Chen S., Wei P., Guo S., Wu J. (2022). A briefly overview of the research progress for the abscisic acid analogues. Front. Chem..

[74] Weng J.K., Ye M., Li B., Noel J.P. (2016). Co-evolution of hormone metabolism and signaling networks expands plant adaptive plasticity. Cell.

[75] Czech Ministry of the Environment (2016). Public Notice No. 153/2016 for the Management of Soil Protection.

[76] Šichorová K., Tlustoš P., Száková J., Kořínek K., Balík J. (2004). Horizontal and vertical variability of heavy metals in the soil of a polluted area. Plant Soil Environ..

[77] Pavlíková D., Zemanová V., Pavlík M. (2023). Health risk and quality assessment of vegetables cultivated on soils from a heavily polluted old mining area. Toxics.

[78] Přerostová S., Dobrev P.I., Knirsch V., Jarošová J., Gaudinová A., Zupková B., Prášil I.T., Janda T., Brzobohatý B., Skalák J. (2021). Light quality and intensity modulate cold acclimation in *Arabidopsis*. Int. J. Mol. Sci..

