# Supplementary material for: The Feedback of Stress Phytohormones in Avena sativa (L.) on Soil Multi-Contamination
Source: Plants (Basel). 2025 Aug 16;14(16):2554. doi: 10.3390/plants14162554 (PMC12388868; doi:10.3390/plants14162554)
Supplement: Supplementary file 1 [file plants-14-02554-s001.zip › Table S3.pdf]

Table S3 Correlation coefficients of phytohormones in the leaves and roots of oat

**Leaves**														
	ABA	ABA-GE	PA	DPA	NeoPA	ABA met	JA	JA-Ile	JA-Me	DiH-JA	JA met	bJAs	SA	SAG
ABA		*0.322*	***0.011***	*0.057*	***0.004***	*0.844*	***0.001***	***0.001***	***0.003***	***0.001***	***0.001***	***0.001***	***0.004***	*0.098*
ABA-GE	-0.403		*0.060*	*0.170*	*0.320*	*0.056*	*0.069*	*0.062*	***0.044***	*0.075*	*0.060*	*0.066*	*0.052*	*0.547*
PA	**0.828**	-0.687		*0.063*	***0.049***	*0.909*	***0.003***	***0.008***	***0.001***	***0.000***	***0.004***	***0.004***	***0.007***	*0.311*
DPA	-0.692	0.537	-0.680		***0.015***	*0.298*	***0.012***	***0.008***	***0.008***	***0.028***	***0.010***	***0.010***	***0.008***	***0.030***
NeoPA	**0.884**	-0.405	**0.709**	**-0.810**		*0.774*	***0.005***	***0.006***	***0.011***	***0.027***	***0.007***	***0.005***	***0.016***	*0.161*
ABA met	0.083	0.694	-0.048	0.422	-0.122		*0.572*	*0.449*	*0.544*	*0.775*	*0.504*	*0.533*	*0.418*	*0.539*
JA	**0.937**	-0.671	**0.889**	**-0.824**	**0.868**	-0.237		***<0.0001***	***<0.0001***	***<0.0001***	***<0.0001***	***<0.0001***	***<0.0001***	*0.068*
JA-Ile	**0.914**	-0.683	**0.845**	**-0.845**	**0.863**	-0.314	**0.995**		***<0.0001***	***0.000***	***<0.0001***	***<0.0001***	***<0.0001***	*0.050*
JA-Me	**0.891**	**-0.720**	**0.938**	**-0.849**	**0.829**	-0.254	**0.983**	**0.971**		***<0.0001***	***<0.0001***	***<0.0001***	***<0.0001***	*0.087*
DiH-JA	**0.915**	-0.660	**0.949**	**-0.762**	**0.765**	-0.121	**0.970**	**0.949**	**0.979**		***<0.0001***	***0.000***	***0.000***	*0.100*
JA met	**0.921**	-0.686	**0.875**	**-0.837**	**0.851**	-0.278	**0.998**	**0.998**	**0.982**	**0.968**		***<0.0001***	***<0.0001***	*0.056*
bJAs	**0.931**	-0.676	**0.878**	**-0.832**	**0.867**	-0.261	**1.000**	**0.997**	**0.981**	**0.965**	**0.999**		***<0.0001***	*0.062*
SA	**0.881**	-0.702	**0.850**	**-0.850**	**0.805**	-0.334	**0.982**	**0.989**	**0.970**	**0.959**	**0.991**	**0.985**		***0.050***
SAG	-0.625	0.252	-0.411	**0.757**	-0.547	0.257	-0.672	-0.707	-0.640	-0.621	-0.694	-0.683	**-0.707**	
**Roots**														
	ABA	ABA-GE	PA	DPA	NeoPA	ABA met	JA	JA-Ile	JA-Me	DiH-JA	JA met	bJAs	SA	SAG
ABA		***0.000***	***<0.0001***	***<0.0001***	***<0.0001***	***<0.0001***	***<0.0001***	***<0.0001***	***<0.0001***	***<0.0001***	***<0.0001***	***<0.0001***	***0.000***	***0.019***
ABA-GE	**0.954**		***0.000***	***0.001***	***0.000***	***0.000***	***<0.0001***	***0.001***	***0.001***	***0.000***	***0.001***	***0.000***	***<0.0001***	*0.091*
PA	**0.994**	**0.961**		***<0.0001***	***<0.0001***	***<0.0001***	***<0.0001***	***<0.0001***	***<0.0001***	***<0.0001***	***<0.0001***	***<0.0001***	***0.000***	***0.024***
DPA	**0.997**	**0.933**	**0.993**		***<0.0001***	***<0.0001***	***<0.0001***	***<0.0001***	***<0.0001***	***<0.0001***	***<0.0001***	***<0.0001***	***0.001***	***0.013***
NeoPA	**0.987**	**0.946**	**0.993**	**0.992**		***<0.0001***	***<0.0001***	***<0.0001***	***<0.0001***	***<0.0001***	***<0.0001***	***<0.0001***	***0.001***	***0.026***
ABA met	**0.998**	**0.946**	**0.996**	**0.999**	**0.994**		***<0.0001***	***<0.0001***	***<0.0001***	***<0.0001***	***<0.0001***	***<0.0001***	***0.001***	***0.016***
JA	**0.986**	**0.983**	**0.990**	**0.977**	**0.981**	**0.984**		***<0.0001***	***<0.0001***	***<0.0001***	***<0.0001***	***<0.0001***	***<0.0001***	***0.033***
JA-Ile	**0.974**	**0.936**	**0.991**	**0.978**	**0.979**	**0.981**	**0.973**		***<0.0001***	***<0.0001***	***<0.0001***	***<0.0001***	***0.002***	***0.022***
JA-Me	**0.981**	**0.932**	**0.994**	**0.989**	**0.993**	**0.990**	**0.975**	**0.994**		***<0.0001***	***<0.0001***	***<0.0001***	***0.002***	***0.017***
DiH-JA	**1.000**	**0.952**	**0.994**	**0.998**	**0.991**	**0.999**	**0.985**	**0.974**	**0.983**		***<0.0001***	***<0.0001***	***0.000***	***0.019***
JA met	**0.979**	**0.940**	**0.993**	**0.982**	**0.982**	**0.985**	**0.976**	**1.000**	**0.995**	**0.978**		***<0.0001***	***0.002***	***0.022***
bJAs	**0.983**	**0.956**	**0.997**	**0.984**	**0.986**	**0.988**	**0.986**	**0.998**	**0.994**	**0.983**	**0.998**		***0.001***	***0.024***
SA	**0.954**	**0.986**	**0.943**	**0.929**	**0.931**	**0.940**	**0.969**	**0.899**	**0.904**	**0.952**	**0.906**	**0.925**		*0.087*
SAG	**0.793**	0.634	**0.775**	**0.817**	**0.769**	**0.803**	**0.747**	**0.780**	**0.801**	**0.792**	**0.783**	**0.775**	0.641	

The upper part of the table displays the *p*-values (in italics) corresponding to the Pearson's correlation coefficients presented in the lower part of the table. Numbers in bold and red font indicate statistically significant results.
