# Supplementary material for: The Feedback of Stress Phytohormones in Avena sativa (L.) on Soil Multi-Contamination
Source: Plants (Basel). 2025 Aug 16;14(16):2554. doi: 10.3390/plants14162554 (PMC12388868; doi:10.3390/plants14162554)
Supplement: Supplementary file 1 [file plants-14-02554-s001.zip › Table S1.pdf]

Table S1 Accumulation of Cd, Pb, Zn and dry biomass of oat leaves and roots

Parameter	leaves		roots	
	Control	Multi-contamination	Control	Multi-contamination
Cd (mg kg^{-1} DW)	<0.1	6.3 ± 0.7	<0.1	16.6 ± 1.6
Pb (mg kg^{-1} DW)	<2	23.5 ± 3.3	<2	120.2 ± 5.7
Zn (mg kg^{-1} DW)	39.1 ± 1.9	77.5 ± 3.4	51.8 ± 4.0	106.6 ± 5.4
DW (g plant^{-1})	0.086 ± 0.009	0.058 ± 0.004	0.020 ± 0.004	0.019 ± 0.003

DW – dry weight
