# Supplementary material for: The Feedback of Stress Phytohormones in Avena sativa (L.) on Soil Multi-Contamination
Source: Plants (Basel). 2025 Aug 16;14(16):2554. doi: 10.3390/plants14162554 (PMC12388868; doi:10.3390/plants14162554)
Supplement: Supplementary file 1 [file plants-14-02554-s001.zip › Table S2.pdf]

Table S2 Stress phytohormones and their metabolites in the leaves and roots of oat

Parameter	leaves		roots	
	Control	Multi-contamination	Control	Multi-contamination
JA (pmol g^{-1} FW)	43.7 ± 10.3	572.4 ± 43.9	708.5 ± 39.0	234.3 ± 32.2
JA-Ile (pmol g^{-1} FW)	13.4 ± 1.6	244.3 ± 30.1	1329.1 ± 198.3	223.7 ± 10.8
JA-Me (pmol g^{-1} FW)	2.7 ± 0.8	16.5 ± 0.9	16.5 ± 1.8	3.4 ± 0.3
DiH-JA (pmol g^{-1} FW)	14.2 ± 6.6	68.1 ± 5.5	118.1 ± 5.0	12.2 ± 1.2
SA (pmol g^{-1} FW)	161.4 ± 14.7	724.9 ± 73.8	3685.1 ± 585.4	1205.7 ± 104.1
SAG (pmol g^{-1} FW)	5591.0 ± 247.6	5131.6 ± 346.8	1116.9 ± 158.2	805.9 ± 135.0
ABA (pmol g^{-1} FW)	29.5 ± 2.6	42.7 ± 4.1	138.7 ± 7.0	15.0 ± 1.4
ABA-GE (pmol g^{-1} FW)	71.7 ± 7.1	59.3 ± 7.4	39.7 ± 6.8	8.4 ± 1.4
PA (pmol g^{-1} FW)	22.9 ± 1.1	38.6 ± 5.6	37.9 ± 2.2	6.2 ± 0.1
DPA (pmol g^{-1} FW)	26.4 ± 3.6	19.1 ± 1.3	359.0 ± 28.0	33.4 ± 2.8
NeoPA (pmol g^{-1} FW)	0.4 ± 0.05	0.5 ± 0.06	0.7 ± 0.05	0.2 ± 0.01

FW – fresh weight
